# Sex differences in the SOFA score of ICU patients with sepsis or septic shock: a nationwide analysis

**DOI:** 10.1186/s13054-024-04996-y

**Published:** 2024-06-27

**Authors:** Tobias Zimmermann, Philip Kaufmann, Simon A. Amacher, Raoul Sutter, Gregor Loosen, Hamid Merdji, Julie Helms, Atanas Todorov, Pimrapat Gebert, Vera Regitz-Zagrosek, Catherine Gebhard, Mervyn Singer, Martin Siegemund, Caroline E. Gebhard, the Swiss ICU Trial group

**Affiliations:** 1 Intensive Care Unit, Department of Acute Medicine, University Hospital Basel, Petersgraben 4, 4031 Basel, Switzerland; 2 Bloomsbury Institute of Intensive Care Medicine, University College London, London, UK; 3 University of Basel, Basel, Switzerland; 4 Université de Strasbourg (UNISTRA), Hôpitaux Universitaires de Strasbourg, Service de Médecine Intensive-Réanimation, Faculté de Médecine, Nouvel Hôpital Civil, Strasbourg, France; 5 INSERM (French National Institute of Health and Medical Research), UMR 1260, Regenerative Nanomedicine (RNM), FMTS, Strasbourg, France; 6 Department of Nuclear Medicine, University Hospital Zurich, Zurich, Switzerland; 7 Center for Molecular Cardiology, University of Zurich, Zurich, Switzerland; 8 Institute of Biometry and Clinical Epidemiology, Charité - Universitätsmedizin Berlin, Berlin, Germany; 9 University of Zurich, Zurich, Switzerland; 10 Institute of Gender in Medicine (GiM), Charité - Universitätsmedizin Berlin, Berlin, Germany; 11 Department of Cardiology, Inselspital Bern, Bern, Switzerland

**Keywords:** Sex differences, Disparities, Infectious diseases, Sepsis, Risk assessment, SOFA score

## Abstract

**Background:**

The Sequential Organ Failure Assessment (SOFA) score is an important tool in diagnosing sepsis and quantifying organ dysfunction. However, despite emerging evidence of differences in sepsis pathophysiology between women and men, sex is currently not being considered in the SOFA score. We aimed to investigate potential sex-specific differences in organ dysfunction, as measured by the SOFA score, in patients with sepsis or septic shock and explore outcome associations.

**Methods:**

Retrospective analysis of sex-specific differences in the SOFA score of prospectively enrolled ICU patients with sepsis or septic shock admitted to one of 85 certified Swiss ICUs between 01/2021 and 12/2022.

**Results:**

Of 125,782 patients, 5947 (5%) were admitted with a clinical diagnosis of sepsis (2244, 38%) or septic shock (3703, 62%). Of these, 5078 (37% women) were eligible for analysis. A statistically significant difference of the total SOFA score on admission was found between women (mean 7.5 ± SD 3.6 points) and men (7.8 ± 3.6 points, Wilcoxon rank-sum *p* < 0.001). This was driven by differences in the coagulation (*p* = 0.008), liver (*p* < 0.001) and renal (*p* < 0.001) SOFA components. Differences between sexes were more prominent in younger patients < 52 years of age (women 7.1 ± 4.0 points vs men 8.1 ± 4.2 points, *p* = 0.004). No sex-specific differences were found in ICU length of stay (women median 2.6 days (IQR 1.3–5.3) vs men 2.7 days (IQR 1.2–6.0), *p* = 0.13) and ICU mortality (women 14% vs men 15%, *p* = 0.17).

**Conclusion:**

Sex-specific differences exist in the SOFA score of patients admitted to a Swiss ICU with sepsis or septic shock, particularly in laboratory-based components. Although the clinical meaningfulness of these differences is unclear, a reevaluation of sex-specific thresholds for SOFA score components is warranted in an attempt to make more accurate and individualised classifications.

**Graphical abstract:**

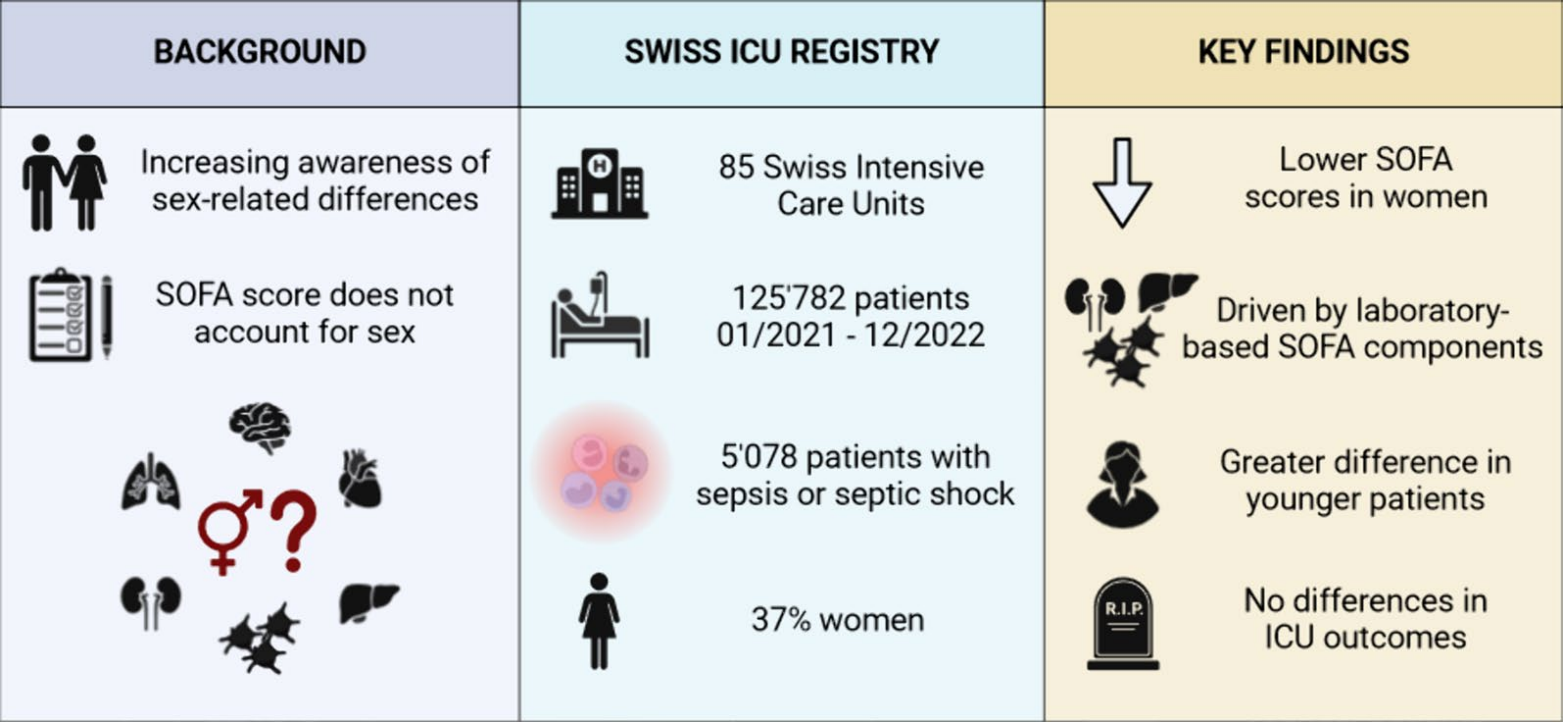

**Supplementary Information:**

The online version contains supplementary material available at 10.1186/s13054-024-04996-y.

## Introduction

Sepsis is defined as life-threatening organ dysfunction caused by a dysregulated host response to infection [1]. To quantify the degree of organ dysfunction, the Sepsis-related Organ Failure Assessment (SOFA) score was developed by a panel of experts and first published in 1996 [2]. The SOFA score consists of six components, each representing an organ system: neurological, cardiovascular, respiratory, renal, liver and coagulation. The SOFA score has since been applied more widely across a variety of critical conditions and the name of the score was accordingly changed to Sequential Organ Failure Assessment score. This score has become an integral part of the daily monitoring of patients in intensive care units (ICU) in Switzerland and worldwide, and is often used as an outcome measure in clinical studies [3].

At the time of initial publication of the SOFA score, knowledge about sex and gender differences was scarce. While sex refers to the biological differences between men and women defined by genes, sex hormones, and anatomy, gender encompasses the sociocultural roles, relations and behaviours [4]. Over the last decade, gender medicine, encompassing both sex and gender, has increasingly gained interest in critical care research [5, 6]. Recent research has aimed to investigate sex and gender differences in overall physiology, disease pathogenesis, provision of ICU resources, and outcomes [3, 7, 8]. Biological differences within the immune response and its modification by sex steroids have been discussed as a potential contributor to the increased susceptibility to infection and sepsis described in men [6, 9]. Despite calls for more personalized risk stratification and decision making in clinical practice, most ICU illness severity scores and estimates of organ dysfunction lack sex-specific thresholds. This may potentially lead to an inaccurate reflection of the disease state, limiting their validity [10]. Existing literature on sex differences in the SOFA score is limited to small single centre patient populations and specific diagnostic groups [10, 11]. The SOFA score is currently in the process of being updated and potentially extended to better encompass contemporary organ support techniques and improve accuracy [12]. However, the importance of considering biological sex remains unclear. Thus, we aimed to investigate sex-specific differences in organ dysfunction, as measured by the SOFA score, in patients with sepsis or septic shock. Secondary aims included describing the cohort and investigating the association between SOFA score and ICU outcomes such as ICU mortality and length-of-stay.

## Methods

### Database and data validation

This study is based on the Swiss ICU-registry (MDSi—Minimal Dataset for ICUs) of the Swiss Society of Intensive Care Medicine (SSICM), a mandatory database containing prospectively collected and validated information about all patients admitted to the 85 certified ICUs in Switzerland. After initial data entry, the data are first locally validated by staff physicians after which the anonymized data are transferred to a secure central database, as previously described [8, 13]. During the import process into the central database, all MDSi data including SOFA scores are validated according to several criteria defined in the MDSi manual [14]. In case of detected outliers or missing data, the respective ICU is contacted and asked to review and potentially revise the data. In 2016, the basic dataset was extended by information regarding advance directives/treatment limitations [15] and in January 2021, the SOFA score became an obligatory addition to the dataset [14].

### Variables

Information was extracted on demographics, origin and type of admission, admission diagnosis, and the admission Simplified Acute Physiology Score (SAPS II) [16], ICU length of stay (LOS) and discharge details including ICU mortality. For a maximum of 21 days after admission, daily SOFA scores including organ-specific scores, process variables comprising interventions before and during ICU stay, nursing workload and treatment modalities such as ventilatory support, vasopressor use, renal replacement therapy (RRT) according to the Nine Equivalents of Nursing Manpower Use Score (NEMS) [17], and information about delirium identified by the Sedation Agitation Scale (SAS) were also extracted. Interventions in the ICU included but were not limited to procedures such as endotracheal intubation, pacemaker insertion, cardioversion and endoscopy.

### SOFA score calculation

For the Swiss ICU-registry, daily SOFA scores are calculated by physicians (for the previous 24 h, 07:00 am–07:00 am) and include the total score as well as scores from each of the six organ-specific components. In all participating ICUs, the calculation of the SOFA score is performed according to the guidelines released by the Swiss Society of Intensive Care Medicine [14]. In the specific case of ICU stays < 24 h, the SOFA score is calculated based on data from ICU admission to discharge, incorporating data (e.g., laboratory values) from 07:00 am on the day of ICU admission. If necessary, data will be extrapolated based on data recorded during ICU stay (e.g. urine output). If a pathological laboratory value is not re-measured, the value can be carried forward for a maximum of 72 h. A value of 0 is assigned if the SOFA component is within the normal range, never determined, or not re-determined after 72 h. Facing the well-established challenge of Glasgow Coma Scale (GSC) calculation in sedated and intubated patients in the ICU, the assumed (if the patient did not receive sedation) or last known GCS is used.

### Ethics

This study was approved by the Ethics Committee of Northwestern and Central Switzerland (EKNZ UBE-15/47) and the scientific committee of the SSICM. The study was carried out according to the principles of the Declaration of Helsinki. Reporting is in accordance with STROBE reporting guidelines for observational studies.

### Study population

Patients aged ≥ 16 years admitted to one of the 85 certified ICUs in Switzerland between 1st January 2021 and 31st December 2022 were included in the study. All patients in whom sepsis or septic shock was recorded as the main admission diagnosis were identified electronically. Patients who did not meet the minimum sepsis criteria at admission according to Sepsis-3 guidelines with a first recorded SOFA score ≥ 2 were excluded [1]. Supplementary Fig. 1 depicts patient selection from the MDSi dataset.

### Statistical methods

Continuous variables are presented as mean ± standard deviation (SD) or median with interquartile range (IQR) for highly skewed variables. Categorical variables are expressed as counts and proportions. Wilcoxon rank-sum test was applied for comparisons between two continuous variables, Pearson’s Chi-squared test without continuity correction was used for comparisons of categorical variables. State occupancy plots were used to display the dynamic distribution of total SOFA and organ-specific scores over time. Competing states, such as discharge or death were carried forward to avoid bias. To relax linearity assumptions in logistic regression models, continuous variables and SOFA scores were fitted with restricted cubic splines and four knots each [18]. Analysis of variance (ANOVA) was used to understand if there was a significant interaction between independent variables. Restricted interactions, dropping doubly non-linear interaction terms, were used for interactions between variables modelled with splines. Conditional effect plots are utilized to show the association between the covariate of interest and the outcome, holding other covariates constant at the median if continuous, mode if categorical, or lowest value if binary. Several sensitivity analyses were performed: All patients with an advance directive were excluded to test for potential bias in ICU mortality introduced by treatment limitations. Patients with a post-operative/-interventional ICU admission were excluded to investigate differences in an emergency admission population only. Given the potential impact of sex hormones on the immune response and a potential biological advantage in women, patients were stratified above and below 52 years of age, the median age of menopause for women in Switzerland [19].

There were no missing data in any of the variables used for this analysis, since a complete dataset is mandatory for a patient to be entered into the Swiss ICU-registry. As this study was a retrospective data analysis, no power calculation was performed. All results are hypothesis-generating and no causality is assumed with any of the findings. All hypothesis testing was two-tailed, with *p* values < 0.05 considered statistically significant. All analyses were performed using R version 4.3.1 [20].

## Results

### Study population

Over the two-year study period, 125,782 patients were admitted to an ICU in Switzerland. Of these, 5947 (5%) were admitted with a diagnosis of sepsis (2244, 38%) or septic shock (3703, 62%), with a total of 5078 patients being eligible for analysis (Supplementary Fig. 1). Of the eligible patients, 37% (n = 1901) were women. Sixty-five percent (n = 1236) of women were admitted with a diagnosis of septic shock compared to 63% (n = 2009) of men. The mean (± SD) age in this cohort was 69 ± 13 years in men and 67 ± 15 years in women (*p* = 0.18) (Table 1). 1710 (34%) patients (32% men, 37% women) had surgery or another intervention before ICU admission, with the most frequent sites being gastrointestinal 671 (13%), orthopaedic 230 (4.5%) and urological 197 (3.9%). Seven women (0.4%) had a gynaecological or obstetric surgery/intervention (Table 1).

**Table 1:** Baseline characteristics stratified by sex

	Overall N = 5078	Men N = 3177	Women N = 1901	*p* value^a^
Admission diagnosis, n (%)				0.20
Sepsis	1833 (36)	1168 (37)	665 (35)	
Septic shock	3245 (64)	2009 (63)	1236 (65)	
Age (years), Mean (SD)	68.2 (14.0)	68.7 (13.1)	67.4 (15.2)	0.18
ICU admission from, n (%)				0.083
Emergency Department	2038 (40)	1294 (41)	744 (39)	
Ward	1398 (28)	866 (27)	532 (28)	
Other ICU	251 (4.9)	161 (5.1)	90 (4.7)	
IMC/recovery room	326 (6.4)	222 (7.0)	104 (5.5)	
Postinterventional	892 (18)	530 (17)	362 (19)	
Other	173 (3.4)	104 (3.3)	69 (3.6)	
Surgery/interventions before ICU admission, n (%)				< 0.001
No intervention	3368 (66)	2161 (68)	1207 (63)	
Gastrointestinal	671 (13)	384 (12)	287 (15)	
Orthopedics	230 (4.5)	145 (4.6)	85 (4.5)	
Urology	197 (3.9)	111 (3.5)	86 (4.5)	
Cardiovascular	94 (1.9)	65 (2.0)	29 (1.5)	
Head and neck	51 (1.0)	34 (1.1)	17 (0.9)	
Gynaecology/obstetrics	7 (0.1)	0 (0)	7 (0.4)	
Other	460 (9.1)	277 (8.7)	183 (9.6)	
Total NEMS normalized to shifts, Mean (SD)	24.4 (6.5)	24.5 (6.7)	24.3 (6.3)	0.36
NEMS of first shift, Mean (SD)	25.6 (8.5)	25.9 (8.6)	25.3 (8.3)	0.040
Mechanical ventilation, n (%)	2367 (47)	1500 (47)	867 (46)	0.27
Vasoactive drug use, n (%)	4016 (79)	2494 (79)	1522 (80)	0.19
Multiple vasoactive drugs, n (%)	1,109 (22)	732 (23)	377 (20)	0.007
Renal replacement therapy, n (%)	631 (12)	416 (13)	215 (11)	0.062
Delirium (as per SAS scale), n (%)	585 (12)	406 (13)	179 (9.4)	< 0.001
Interventions on ICU, n (%)	1910 (38)	1242 (39)	668 (35)	0.005
Non-invasive ventilation, n (%)	4373 (86)	2724 (86)	1649 (87)	0.32
Limitation of treatment, n (%)				0.39
None	3472 (68)	2185 (69)	1287 (68)	
Present at admission	860 (17)	522 (16)	338 (18)	
Put in place during ICU stay	680 (13)	433 (14)	247 (13)	
Put in place upon ICU discharge	66 (1.3)	37 (1.2)	29 (1.5)	
Total SOFA score on admission, Mean (SD)	7.7 (3.6)	7.8 (3.6)	7.5 (3.6)	< 0.001
Maximum total SOFA, Mean (SD)	8.8 (4.0)	9.0 (4.0)	8.5 (4.0)	< 0.001
SAPS II, Mean (SD)	47.7 (18.9)	48.1 (18.8)	47.1 (19.0)	0.041
ICU length of stay (days), Median (IQR)	2.6 (1.3–5.8)	2.7 (1.2–6.0)	2.6 (1.3–5.3)	0.13
ICU mortality, n (%)	745 (15)	483 (15)	262 (14)	0.17

*ICU* intensive care unit, *IMC* intermediate care unit, *IQR* interquartile range, *NEMS* Nine Equivalents of Nursing Manpower Use Score, *LOS* length-of-stay, *RRT* renal replacement therapy, *SAPS* II Simplified Acute Physiology Score II, *SAS* sedation agitation scale, *SOFA* sequential organ failure assessment

^a^Pearson’s Chi-squared test for comparisons between categorical variables; Wilcoxon rank sum test for comparisons between continuous variables

### Total SOFA, max SOFA and SAPS II

**Fig. 1:**
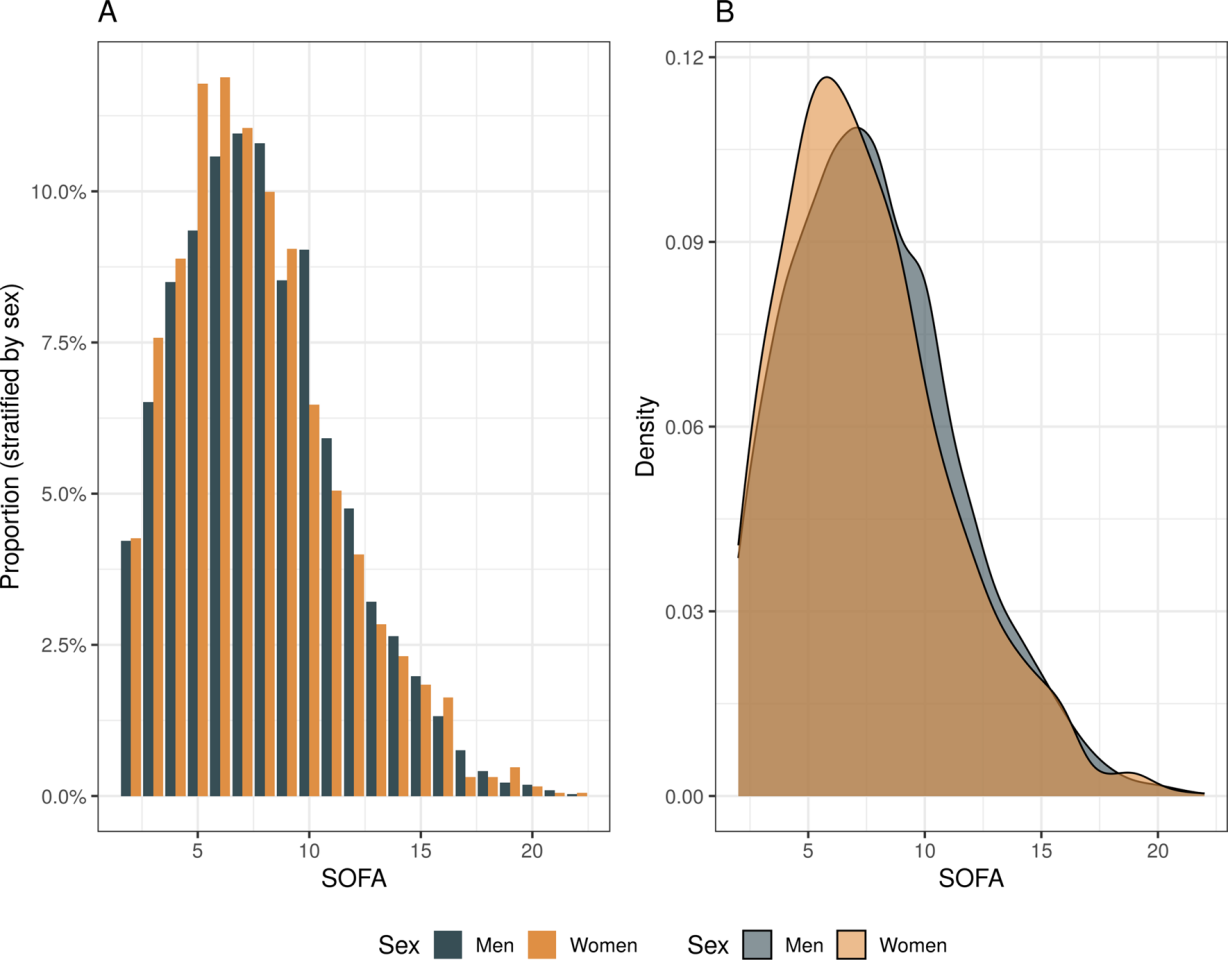
Distribution of total SOFA scores at ICU admission stratified by sex. Histogram (**A**) and density plot (**B**) show a higher proportion of lower scores in women compared to men. *SOFA Sequential Organ Failure Assessment*

In the overall cohort, SOFA scores ranged between 2 and 22 points at admission. Women had a lower admission SOFA score (7.5 ± 3.6 points) compared to men (7.8 ± 3.6 points, *p* < 0.001) (Table 1, Fig. 1). This difference was greater in younger patients when stratifying by age < 52 years (women 7.1 ± 4.0 vs men 8.1 ± 4.2, *p* = 0.004) (Table 2). The mean maximum SOFA score during the first 21 days of ICU stay was 8.8 ± 4.0 points; women (8.5 ± 4.0) had lower maximum scores than men (9.0 ± 4.0, *p* < 0.001) (Supplementary Fig. 2A). Most patients (66%) had their highest SOFA score on admission (Supplementary Fig. 2B). Women had a lower SAPS II at admission (47.1 ± 19.0) compared to men (48.1 ± 18.8, *p* = 0.041) (Table 1).

**Table 2:** Selected baseline characteristics stratified by sex and age

Characteristic	< 52 years				≥ 52 years			
	Overall N = 562	Men N = 308	Women N = 254	*p* value^a^	Overall N = 4,516	Men N = 2,869	Women N = 1,647	*p* value^a^
Age (years), mean (SD)	39.5 (9.7)	40.8 (9.0)	37.8 (10.3)	0.001	71.8 (9.6)	71.7 (9.4)	72.0 (9.8)	0.25
Total SOFA on admission, mean (SD)	7.6 (4.1)	8.1 (4.2)	7.1 (4.0)	0.004	7.7 (3.5)	7.8 (3.5)	7.6 (3.5)	0.011
Maximum total SOFA, mean (SD)	8.7 (4.6)	9.1 (4.6)	8.1 (4.7)	0.004	8.8 (4.0)	9.0 (4.0)	8.6 (3.9)	< 0.001
ICU length of stay (days), median (IQR)	2.7 (4.6)	3.0 (5.9)	2.3 (3.6)	0.056	2.6 (4.5)	2.7 (4.7)	2.6 (4.0)	0.35
ICU mortality, n (%)	40 (7.1)	25 (8.1)	15 (5.9)	0.31	705 (16)	458 (16)	247 (15)	0.39

*ICU* intensive care unit, *IQR* interquartile range, *SOFA* sequential organ failure assessment

^a^Pearson's Chi-squared test for comparisons between categorical variables; Wilcoxon rank sum test for comparisons between continuous variables

### SOFA sub-scores

**Fig. 2:**
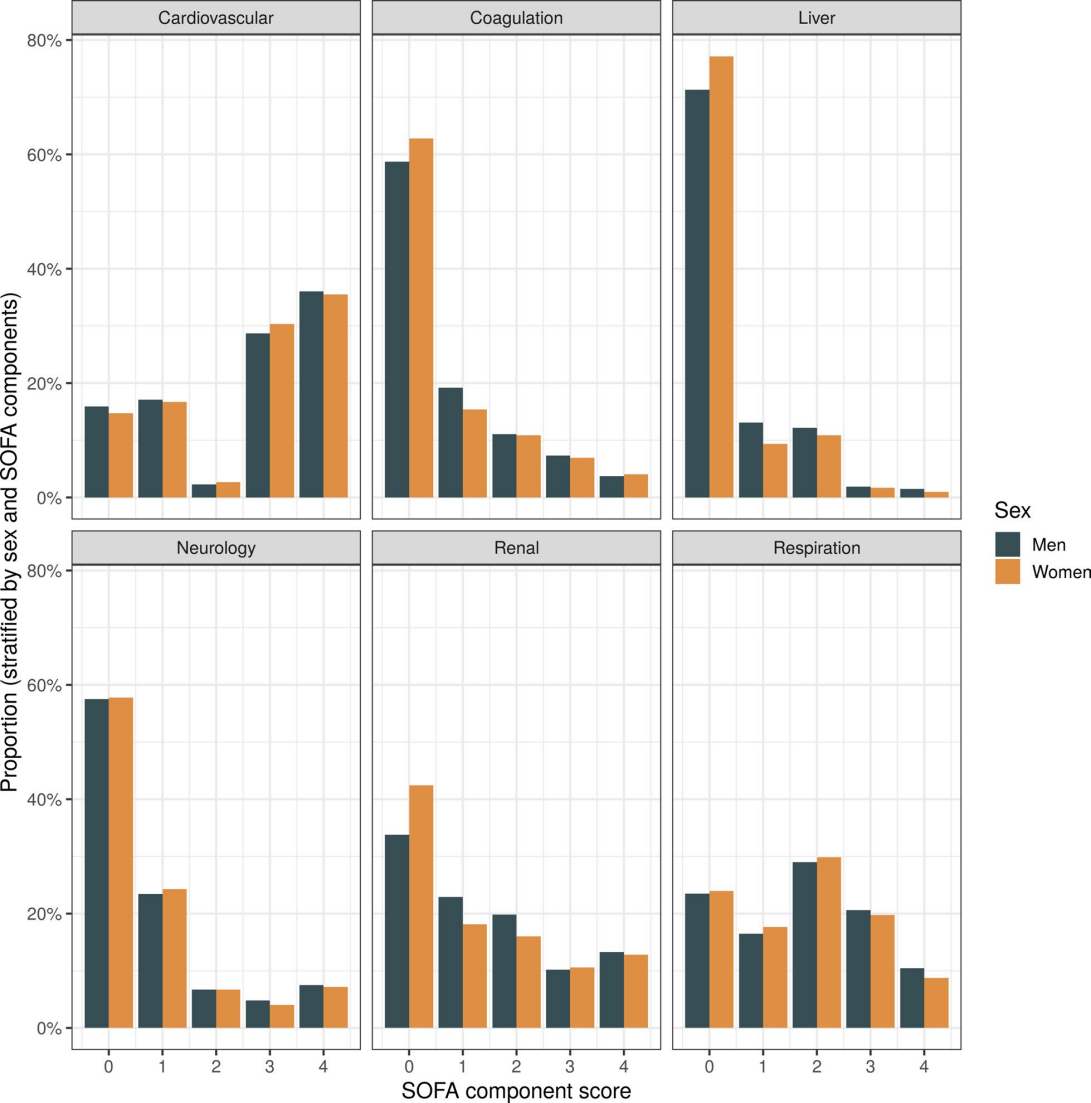
Distribution of SOFA components stratified by sex. Distributions are mostly skewed with a predominance of lower scores with the cardiovascular and respiratory components being the exception. Notable differences between women and men can be observed in coagulation, liver and renal sub-scores, with a higher proportion of women scoring 0 points. *SOFA Sequential Organ Failure Assessment*

The observed distribution of the individual SOFA sub-scores was mostly irregular and skewed, especially in the coagulation, liver and neurological components with a strong predominance of lower scores (Fig. 2). Women more often scored 0 points in the coagulation, liver and renal components (Fig. 2, Supplementary Table 1). Differences between sexes were greatest in younger patients when stratifying the cohort by 52 years of age (Supplementary Fig. 3). Within the cardiovascular component, two points were rarely scored due to minimal use of either dobutamine or dopamine. Compared to other SOFA sub-scores, the cardiovascular component was the only one with an increased prevalence of higher scores compared to lower scores (left-skewed), due to frequent use of vasopressors (Fig. 2).

### Excluding post-operative/-interventional admissions

Excluding 1710 (34%) patients who were admitted to ICU after an operation/intervention, left 3368 (66%) patients available for this sensitivity analysis. Differences in SOFA scores between women (7.4 ± 3.6) and men (7.7 ± 3.6, *p* = 0.006) were similar to the full cohort. There were no sex-specific differences in ICU LOS (*p* = 0.23) and ICU mortality (*p* = 0.22) (Supplementary Table 2).

### Temporal trends of SOFA and SOFA sub-scores

**Fig. 3:**
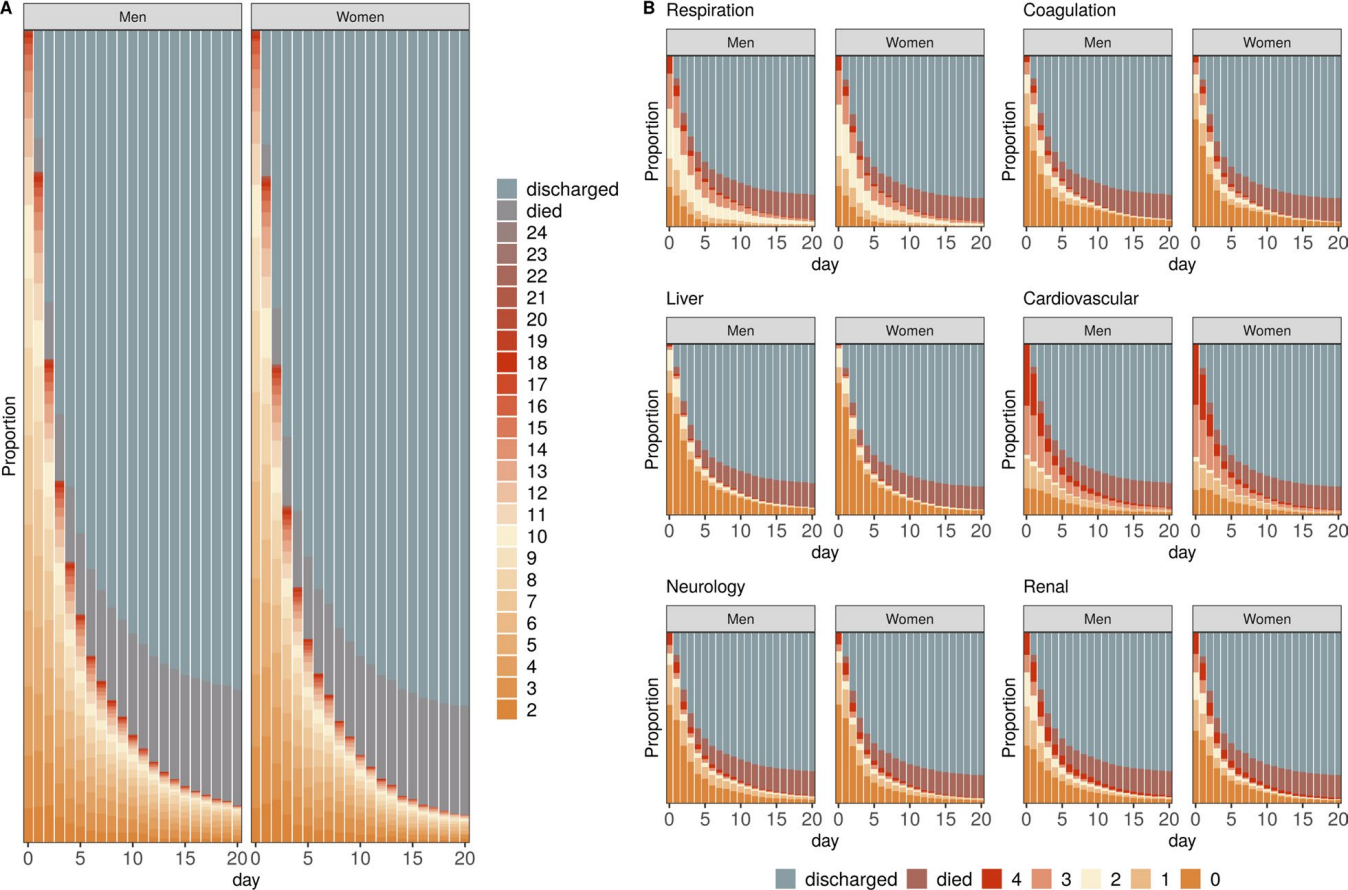
State occupancy plots showing the proportion of patients with a certain SOFA score (y-axis) over time (days, x-axis). For death or discharge from ICU within 21 days, this state was carried forward. **A** The total SOFA score, ranging from 2 to 24 points, over time, **B** SOFA component scores, ranging from 0 to 4 points, over time. *SOFA Sequential Organ Failure Assessment*

The temporal trends of SOFA and its components are demonstrated in the form of state occupancy proportion plots (Fig. 3). In the liver sub-score, most patients scored 0 points with only small variations over time. The cardiovascular component had the largest proportion of high scores (3 and 4 points) on admission, but within a few days there were rapid improvements as reflected by an increasing proportion of lower scores (Fig. 3B). No obvious sex-specific differences were observed in this analysis.

**Fig. 4:**
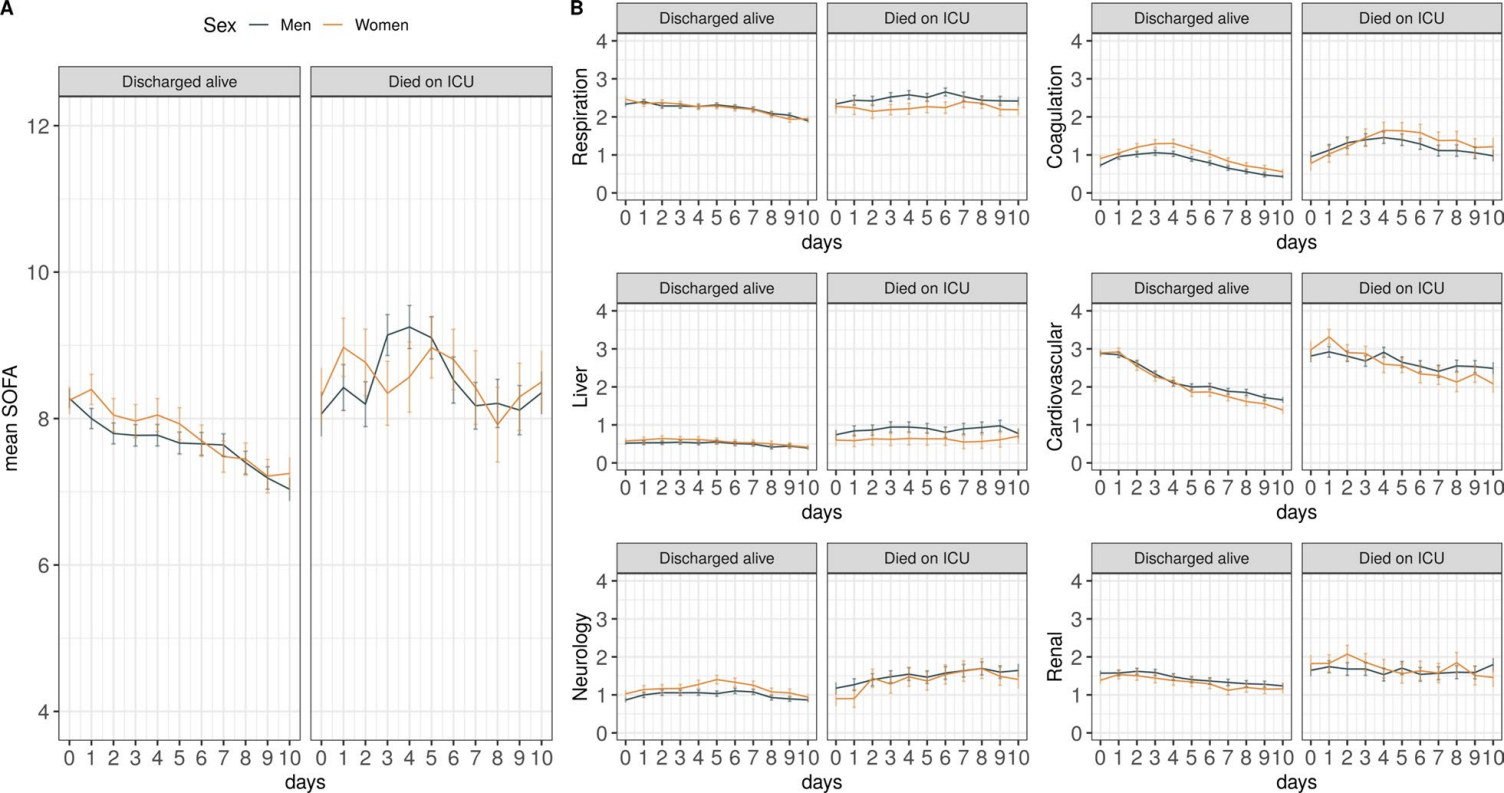
Longitudinal trend of mean total SOFA scores with standard error bars (**A**) and individual component scores (**B**) stratified by sex over a period of 10 days in a subset of patients who were on the ICU for at least 10 days. *SOFA Sequential Organ Failure Assessment*

In a subgroup of patients who stayed on ICU for ≥ 10 days, ICU survivors had an improved total SOFA score after 1–2 days, whereas ICU non-survivors continued to have elevated SOFA sub-scores (Fig. 4). The most notable temporal change was observed in the cardiovascular component, having the steepest downward slope in recovering patients (Fig. 4B).

### Outcomes

**Fig. 5:**
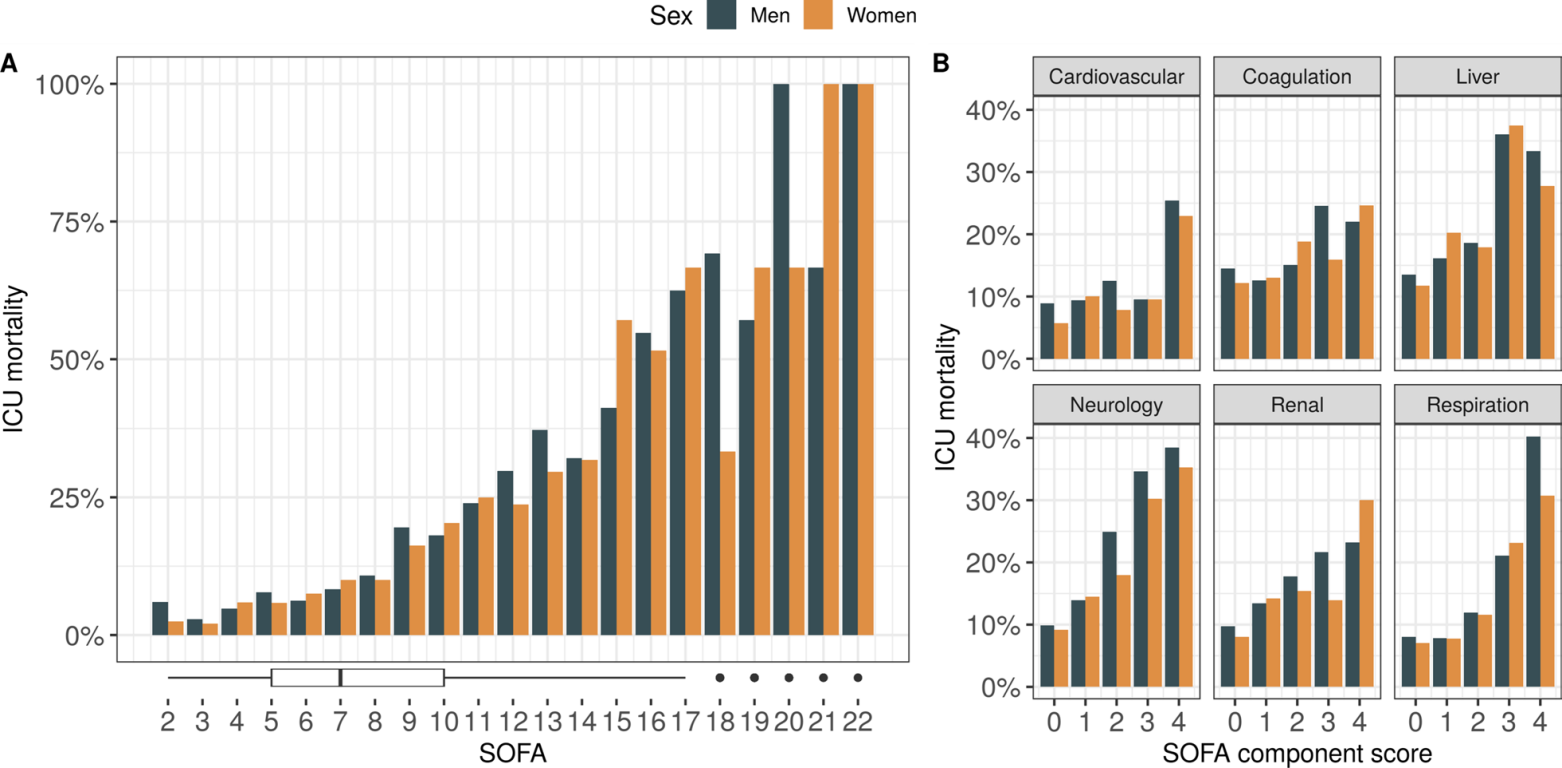
Histogram showing mortality rates in women and men for each of the recorded total SOFA scores at admission (**A**). The boxplot below displays the distribution of SOFA scores in the cohort. In **B**, individual SOFA component scores are plotted against within-group ICU mortality rates. Overall, with few exceptions, higher scores carry a higher mortality risk with no clear differences between women and men. *ICU Intensive Care Unit, SOFA Sequential Organ Failure Assessment*

Median ICU LOS was 2.6 days (IQR 1.3–5.8). ICU mortality was 15%, with no notable sex-specific differences. A higher total SOFA score and individual sub-score at admission was associated with an increased mortality risk (Fig. 5). Mortality was lower in younger patients when stratified by 52 years of age (7% vs. 16%), with no notable sex-specific differences (Table 2). A relevant number of both women (n = 338, 18%) and men (n = 522, 16%) had an advance directive in place upon ICU admission or had one placed during their ICU stay (women n = 247, 13%; men n = 433, 14%) (Table 1). After excluding patients with any form of advance directive, including those who had one put in place upon ICU discharge (n = 1606, 32%), ICU mortality was 6.4%. No difference between sexes was found in ICU mortality (men 6.7% vs women 5.7%, *p* = 0.25) or ICU LOS (men median 2.6 days (IQR 1.2–5.9) vs women 2.6 days (IQR 1.4–5.1), *p* = 0.43) (Supplementary Table 3).

### Regression models

In a logistic regression analysis with total SOFA, age and sex as independent variables and ICU mortality as the dependent variable, no significant interactions were found between sex and age (*p* = 0.54) or sex and total SOFA (*p* = 0.77). A significant interaction was observed, however, between age and total SOFA (*p* = 0.009).

Conditional effect plots of the model showed only a small effect of sex on probabilities for ICU mortality. While age showed a close to linear association, total SOFA was associated with ICU mortality in a non-monotonic fashion, with an almost flat slope up to around 7 points, transforming into a steep upwards slope afterwards (Supplementary Fig. 4A).

In the model including SOFA components, age and sex, no significant interactions were found for sex or age and the SOFA components. Conditional effect plots revealed an almost linear relationship between SOFA components and ICU mortality, with the steepest slopes for the neurological and respiratory components (Supplementary Fig. 4B, Supplementary Fig. 5).

Plotting a quantile regression to the median of age and total SOFA on admission, stratified by sex, showed that while women generally have lower SOFA scores than men, an increase in age is associated with an increase in total SOFA across the whole age-range. Opposite to that, men—following the women’s trend initially—were shown to present with lower SOFA scores from the age of 60 and older (Supplementary Fig. 6).

### Treatments and interventions

Men required a more intense level of nursing care during the first shift after ICU admission, as measured by the NEMS score, (NEMS: men 25.9 ± 8.6 points vs women 25.3 ± 8.3 points, *p* = 0.040). ICU interventions such as mechanical and non-invasive ventilation (NIV) and renal replacement therapy (RRT) did not differ between sexes, however men were treated more frequently with multiple vasoactive agents (23% vs. 20%, *p* = 0.007) and received more interventions during their ICU stay (39% vs. 35%, *p* = 0.005). Compared to women, a larger proportion of men developed delirium (13% vs. 9%, *p* < 0.001) (Table 1).

## Discussion

This nationwide observational study including 5078 patients admitted to Swiss ICUs with a diagnosis of sepsis or septic shock aimed to investigate sex-specific differences in organ dysfunction as measured by the SOFA score and associations with ICU outcomes.

We report the following main findings: First, sex-specific differences in the SOFA score were present with women having lower scores compared to men. This was mainly driven by differences in the laboratory-based coagulation, renal and liver components of the SOFA score. Differences were greater in younger patients < 52 years of age. Second, an increase in SOFA score (total or organ-specific) is associated with an increased risk for ICU mortality. Third, the lower degree of organ dysfunction in women, as measured by the SOFA score, did not translate into better short-term outcomes, such as ICU LOS and ICU mortality. Fourth, sensitivity analyses excluding patients with any form of advance directive, or excluding patients with an operation/intervention prior to ICU admission showed similar results to the main cohort.

Given the variety of scores available and the heterogeneity of ICU patients and their illnesses, reported data on sex differences with regard to illness severity are inconsistent [3, 8, 21, 22]. Recent data from large cohort studies have shown that illness severity estimated by SAPS or APACHE scores at ICU admission is higher in women [3, 8]. However, these studies included heterogeneous ICU populations [3], focused on patients with cardiovascular diseases [8] and did not include the SOFA score. In our study, exclusively focussing on patients with sepsis and septic shock, we found lower SOFA scores in women compared to men. While the identified differences in total SOFA score between sexes were numerically not large, this finding should not be discounted on that basis. Since the SOFA score is frequently applied to large populations, even a seemingly minor difference may have substantial implications. For a diagnosis of sepsis only 2 SOFA score points are required and a difference of just one point can already make a relevant difference—if not clinically then certainly from an administrative and study outcome point of view. While more women had an interventional procedure or surgery immediately before ICU admission, a sensitivity analysis excluding those patients showed similar results compared to the main cohort. We found the SOFA components with the most notable sex-specific differences were those based on laboratory values with equal thresholds applied to men and women. Our findings corroborate previous results that found lower SOFA scores in respiratory and renal components in women, as well as lower serum bilirubin and creatinine [23]. Men in general have a higher muscle mass compared to women of comparable size, resulting in higher levels of creatinine [24, 25]. Therefore, using equal creatinine thresholds for men and women may lead to an underestimation of acute kidney injury (AKI) in women [26]. Sex differences in platelet counts also exist with adult women showing higher numbers of platelets, again potentially leading to underestimation of disease severity in women by using uniform thresholds for both sexes [27, 28]. While the literature on sex-specific differences of liver function in sepsis is scarce, sex differences in the aetiology, pathogenesis and outcomes of liver diseases have been described [29, 30]. Sex-related differences in the laboratory components of the sodium-adjusted Model for End-Stage Liver Disease (MELD) score (which includes bilirubin, creatinine, INR and sodium) put women at a disadvantage regarding liver transplantations and waitlist mortality [31]. This underlines the importance of investigating sex-specific thresholds and/or considering female sex in risk and illness severity scores [32].

Besides the potential bias introduced by uniform thresholds in the SOFA score, outdated definitions may also contribute to imprecision. For example, as previously noted and confirmed in this study, very few patients score two points in the cardiovascular component, most likely due to the outdated consideration of dopamine or dobutamine as vasoactive agents to treat sepsis-associated cardiovascular dysfunction [12, 33]. A more contemporary definition of cut-offs might help improve this imbalance. Despite this, we found a strong association between increased SOFA scores and ICU mortality in this cohort.

Although beneficial hormonal and immune profiles in women have been identified as factors underlying a sex-specific advantage in sepsis, outcome data remain conflicting. Some studies report higher short-term mortality in women [34], while others show contrary results [22, 35, 36]. In our study, we found no differences in ICU mortality between women and men with sepsis or septic shock. This aligns with previous studies [23, 37] and a meta-analysis [38] reporting no significant sex differences in short-term mortality in critically ill patients with sepsis and septic shock. Mewes et al. demonstrated in their prospective study enrolling 737 septic patients that men exhibit more severe organ dysfunction according to SOFA and SOFA sub-scores, yet no sex differences were found in 28- and 90-day mortality [23]. Similarly, we found that organ dysfunction and illness severity, as estimated by the SOFA and SAPS II scores, were lower in women but did not translate into better short-term outcomes. This raises the question of whether yet unrecognized factors in women may mitigate their potential biological advantage.

In this study, we found a significant interaction between age and total SOFA for an outcome of ICU mortality but no significant interactions with sex. We furthermore observed differential associations between age and total SOFA depending on sex with increasing SOFA scores in men until the age of 60 years and a downward trend afterwards, whereas increasing age in women was associated with an increase in total SOFA across the entire age spectrum. A possible explanation could be the decrease in oestrogen after menopause and the predominance of yet unidentified negative factors in women thereafter, making them more susceptible to infection and organ failure. Indeed, sex steroids have been shown to influence immune responses, bacterial metabolism, and the expression of virulence factors [9]. For instance, oestrogens have the capacity to directly enhance the production of immunoglobulins by B-lymphocytes, thereby augmenting the humoral immune response in pre-menopausal women [39]. However, due to the limited number of covariates accessible in the ICU-registry, this association could not be investigated further and so this interpretation is purely speculative. However, these findings are in line with a previous study that had shown sex differences in association between age and in-hospital mortality [37].

It has been previously claimed that women are less frequently admitted to ICUs [8, 40] and undertreated compared to men, both before and during ICU admission, despite being equally or even more severely ill [3, 8, 41, 42]. This included less use of mechanical ventilation and renal replacement therapy and earlier discharge from ICU [41]. In our cohort, women less frequently received treatment with multiple vasoactive agents and interventions while in intensive care, yet no differences in ICU mortality or ICU LOS were observed. This aligns with a recent study in 699,535 ICU patients that reported an even lower overall hospital mortality in women despite significantly less organ support compared to men [42]. However, the impact of the sex-imbalance in provision of ICU care on long-term survival remains uncertain at this point. In addition, sex-unspecific instruments to measure illness severity, unconscious bias and gender-related factors may contribute to the differences observed, the latter often not accounted for in concomitant studies [15].

Women are reported to more frequently have advance directives and treatment limitations in place compared to men, potentially introducing bias into mortality reports stratified by sex [43, 44]. In contrast to these reports, we observed no relevant sex-related differences in the prevalence of treatment limitations. Sensitivity analyses excluding all patients with an advance directive revealed no sex differences regarding ICU outcomes. Limitations of medical treatment prior to ICU admission were also not accountable for the sex-related differences in ICU interventions and outcomes [42]. However, the impact of existing advance directives on sex disparities in ICU admission, and thus underrepresentation of female ICU patients in multiple diagnoses [40], remains unclear.

## Limitations

There are several limitations to consider: First, our study is observational and does not provide information on underlying mechanisms. This includes a lack of information about the aetiology of sepsis/septic shock and more detailed information such as laboratory values (e.g., lactate) and microbiological speciation. Second, our selection of patients is based on the admission diagnosis of sepsis or septic shock and the SOFA score. No post-hoc diagnostic adjudication was performed for this registry study. Similarly, calculation of the SOFA score is a manual physician-dependent task and an occasional miscalculation of scores may be possible. Therefore, we cannot exclude the possibility of misclassification of some patients, although this would be equally likely in male and female patients. Third, information on patient demographics, sociocultural factors, chronic conditions, comorbidities, admission criteria and diagnostic values are limited. Thus, we cannot account for a potential impact of baseline variables on our findings. Fourth, the dataset contains no information about long-term outcomes and is limited to ICU data, therefore no comment can be made about post-intensive care sequelae and hospital/long-term mortality can be made.

## Conclusion

Women have lower SOFA scores at ICU admission for sepsis or septic shock compared to men. Uniform thresholds in laboratory components likely contribute to the differences observed. Despite this, no differences in ICU mortality or LOS were found between men and women. Therefore, the clinical relevance of the observed differences remains unknown. Given the progression towards precision medicine, our study should stimulate future considerations of sex-specific thresholds in the derivation and validation of risk scores and clinical research in general.

### Supplementary Information

Supplementary Material 1.

Supplementary Material 2.

## Data Availability

This study is based on the MDSi dataset by the Swiss Society of Intensive Care Medicine (SSICM). Restrictions apply to the availability of this data and any requests must be made to the scientific committee of the SSICM (https://www.sgi-ssmi.ch).

## Abbreviations

AKI: Acute kidney injury
ANOVA: Analysis of variance
APACHE: Acute Physiology and Chronic Health Evaluation
EKNZ: Ethics Committee of Northwestern and Central Switzerland
ICU: Intensive care unit
IQR: Interquartile range
LOS: Length of stay
MDSi: Minimal Dataset for ICUs
MELD: Model for End-Stage Liver Disease
NEMS: Nine Equivalents of Nursing Manpower Use Score
NIV: Non-invasive ventilation
RRT: Renal replacement therapy
SAPS II: Simplified Acute Physiology Score II
SAS: Sedation Agitation Scale
SD: Standard deviation
SOFA: Sequential Organ Failure Assessment
SSICM: Swiss Society of Intensive Care Medicine
STROBE: Strengthening the reporting of observational studies in epidemiology
