# Supplementary material for: Sex differences in the SOFA score of ICU patients with sepsis or septic shock: a nationwide analysis
Source: Crit Care. 2024 Jun 27;28:209. doi: 10.1186/s13054-024-04996-y (PMC11210104; doi:10.1186/s13054-024-04996-y)
Supplement: Supplementary file 1 — Supplementary Material 1. [file 13054_2024_4996_MOESM1_ESM.docx]

STROBE Statement—checklist of items that should be included in reports of observational studies

|  | Item No. | Recommendation | Page  No. | Relevant text from manuscript |
| --- | --- | --- | --- | --- |
| **Title and abstract** | 1 | (*a*) Indicate the study’s design with a commonly used term in the title or the abstract | 1 | Sex differences in the SOFA score of ICU patients with sepsis or septic shock: a nationwide analysis |
|  |  | (*b*) Provide in the abstract an informative and balanced summary of what was done and what was found | 2 | See abstract |
| Introduction | | | |  |
| Background/rationale | 2 | Explain the scientific background and rationale for the investigation being reported | 4-5 | Despite calls for more personalized risk stratification and decision making in clinical practice, current ICU illness severity scores and estimates of organ dysfunction still lack sex-specific thresholds. This may potentially reflect the disease state in women and men inaccurately. See Introduction. |
| Objectives | 3 | State specific objectives, including any prespecified hypotheses | 4-5 | We sought to describe the population of this nationwide study and assess whether the degree of organ failure, as measured by daily SOFA scoring, is represented equally well in women and men admitted to intensive care with sepsis or septic shock. |
| Methods | | | |  |
| Study design | 4 | Present key elements of study design early in the paper | 5-6 | Prospective nationwide study |
| Setting | 5 | Describe the setting, locations, and relevant dates, including periods of recruitment, exposure, follow-up, and data collection | 5-6 | Nationwide analysis of sex-specific differences in the SOFA score in prospectively enrolled patients with sepsis and septic shock admitted to all 81 certified Swiss ICUs between 01/2021 and 12/2022.  See “Database and Variables” and “Study population” |
| Participants | 6 | (*a*) *Cohort study*—Give the eligibility criteria, and the sources and methods of selection of participants. Describe methods of follow-up  *Case-control study*—Give the eligibility criteria, and the sources and methods of case ascertainment and control selection. Give the rationale for the choice of cases and controls  *Cross-sectional study*—Give the eligibility criteria, and the sources and methods of selection of participants | 5 | All patients with sepsis or septic shock aged ≥16 years admitted to any of the 81 certified ICUs in Switzerland were included in the study. See “Database and Variables” and “Study population” in Methods section |
|  |  | (*b*) *Cohort study*—For matched studies, give matching criteria and number of exposed and unexposed  *Case-control study*—For matched studies, give matching criteria and the number of controls per case |  | *Not applicable* |
| Variables | 7 | Clearly define all outcomes, exposures, predictors, potential confounders, and effect modifiers. Give diagnostic criteria, if applicable | 5-6 | The primary aim of this study was to investigate sex-specific differences in the SOFA score and its sub-scores in ICU patients with sepsis and septic shock, and associations with ICU mortality. See “Variables and Outcomes” and “Study population” in Methods section |
| Data sources/ measurement | 8* | For each variable of interest, give sources of data and details of methods of assessment (measurement). Describe comparability of assessment methods if there is more than one group | 5 | See “Database and Variables” in Methods section |
| Bias | 9 | Describe any efforts to address potential sources of bias | 7 | Sensitivity analyses were performed excluding all patients with an advance directive to test for potential bias introduced by treatment limitations. Also see Limitations section. |
| Study size | 10 | Explain how the study size was arrived at | 7 | As this study was a retrospective data analysis based on prospectively collected data, no power calculation was performed. See “Study population” |

Continued on next page

| Quantitative variables | 11 | Explain how quantitative variables were handled in the analyses. If applicable, describe which groupings were chosen and why | 6-7 | Continuous variables are presented as mean ± standard deviation (SD) or median with interquartile range (IQR) for highly skewed variables. Categorical variables are expressed as counts and proportions. |
| --- | --- | --- | --- | --- |
| Statistical methods | 12 | (*a*) Describe all statistical methods, including those used to control for confounding | 6-7 | Wilcoxon rank-sum test was applied for comparisons between two continuous variables, Pearson’s Chi-squared test without continuity correction was used for comparisons of categorical variables. State occupancy plots are used to display the dynamic distribution of total SOFA and organ-system sub-scores over time. Competing states, such as discharge or death were carried forward to avoid bias. Logistic regression models were fit for data description rather than prediction. To relax linearity assumptions, continuous variables and SOFA scores were fitted with restricted cubic splines and four knots each. Conditional effect plots are utilized to show the association between the covariate of interest and the outcome |
|  |  | (*b*) Describe any methods used to examine subgroups and interactions | 6 | Chunk tests for significance of interactions were performed with analysis of variance (ANOVA). |
|  |  | (*c*) Explain how missing data were addressed |  | No missing data were present in the variables used for this analysis. |
|  |  | (*d*) *Cohort study*—If applicable, explain how loss to follow-up was addressed  *Case-control study*—If applicable, explain how matching of cases and controls was addressed  *Cross-sectional study*—If applicable, describe analytical methods taking account of sampling strategy |  | *Not applicable* |
|  |  | (*e*) Describe any sensitivity analyses | 7 | Sensitivity analyses were performed excluding all patients with an advance directive to test for potential bias introduced by treatment limitations. |
| Results | | | | |
| Participants | 13* | (a) Report numbers of individuals at each stage of study—eg numbers potentially eligible, examined for eligibility, confirmed eligible, included in the study, completing follow-up, and analysed | 7 | See “Study population” in Methods and Results Section, Supplementary Figure 1 |
|  |  | (b) Give reasons for non-participation at each stage | 7 | See “Study population” in Methods and Results Section, Supplementary Figure 1 |
|  |  | (c) Consider use of a flow diagram |  | See Supplementary Figure 1 |
| Descriptive data | 14* | (a) Give characteristics of study participants (eg demographic, clinical, social) and information on exposures and potential confounders | 7-8 | See “Study population” and “Treatment and interventions” in Results Section |
|  |  | (b) Indicate number of participants with missing data for each variable of interest |  | 0 |
|  |  | (c) *Cohort study*—Summarise follow-up time (eg, average and total amount) |  | *Not applicable* |
| Outcome data | 15* | *Cohort study*—Report numbers of outcome events or summary measures over time |  | *Not applicable* |
|  |  | *Case-control study—*Report numbers in each exposure category, or summary measures of exposure |  | *Not applicable* |
|  |  | *Cross-sectional study—*Report numbers of outcome events or summary measures |  | *Not applicable* |
| Main results | 16 | (*a*) Give unadjusted estimates and, if applicable, confounder-adjusted estimates and their precision (eg, 95% confidence interval). Make clear which confounders were adjusted for and why they were included | 7-10 | Descriptive analysis. |
|  |  | (*b*) Report category boundaries when continuous variables were categorized |  | *Not applicable* |
|  |  | (*c*) If relevant, consider translating estimates of relative risk into absolute risk for a meaningful time period |  | *Not applicable* |

Continued on next page

| Other analyses | 17 | Report other analyses done—eg analyses of subgroups and interactions, and sensitivity analyses | 7-10 | In a logistic regression analysis with total SOFA, age and sex as independent variables and ICU death as the dependent variable, no significant interactions were found between sex and age or sex and total SOFA. |
| --- | --- | --- | --- | --- |
| Discussion | | | | |
| Key results | 18 | Summarise key results with reference to study objectives | 10-11 | See six main findings at the beginning of Discussion section. |
| Limitations | 19 | Discuss limitations of the study, taking into account sources of potential bias or imprecision. Discuss both direction and magnitude of any potential bias | 13-14 | See Limitations in Discussion Section |
| Interpretation | 20 | Give a cautious overall interpretation of results considering objectives, limitations, multiplicity of analyses, results from similar studies, and other relevant evidence | 14 | The clinical relevance of the observed differences in SOFA score remains unknown at present. Given the progression towards precision medicine, our study should stimulate future considerations of sex- specific thresholds in the derivation and validation of risk scores and clinical research in general. |
| Generalisability | 21 | Discuss the generalisability (external validity) of the study results | 10 | Swiss nationwide observational study including 5’078 patients admitted to Swiss ICUs with a diagnosis of sepsis or septic shock. |
| Other information | |  | | |
| Funding | 22 | Give the source of funding and the role of the funders for the present study and, if applicable, for the original study on which the present article is based | 17 | None |

*Give information separately for cases and controls in case-control studies and, if applicable, for exposed and unexposed groups in cohort and cross-sectional studies.

**Note:** An Explanation and Elaboration article discusses each checklist item and gives methodological background and published examples of transparent reporting. The STROBE checklist is best used in conjunction with this article (freely available on the Web sites of PLoS Medicine at http://www.plosmedicine.org/, Annals of Internal Medicine at http://www.annals.org/, and Epidemiology at http://www.epidem.com/). Information on the STROBE Initiative is available at www.strobe-statement.org.
