# Supplementary material for: Sex differences in the SOFA score of ICU patients with sepsis or septic shock: a nationwide analysis
Source: Crit Care. 2024 Jun 27;28:209. doi: 10.1186/s13054-024-04996-y (PMC11210104; doi:10.1186/s13054-024-04996-y)

**Sex differences in the SOFA score of ICU patients**

**with sepsis or septic shock: a nationwide analysis**

Zimmermann T, Kaufmann P, et al.

**Supplementary Tables and Figures**

**Supplementary Table 1:** SOFA components and their distribution, stratified by sex. *SOFA sequential organ failure assessment*

| **SOFA component** | **Overall**  N = 5,078 | **Men**  N = 3,177 | **Women**  N = 1,901 | **p-value**^1^ |
| --- | --- | --- | --- | --- |
| Respiration, n (%) |  |  |  | 0.24 |
| 0 | 1,201 (24) | 746 (23) | 455 (24) |  |
| 1 | 859 (17) | 523 (16) | 336 (18) |  |
| 2 | 1,489 (29) | 921 (29) | 568 (30) |  |
| 3 | 1,030 (20) | 654 (21) | 376 (20) |  |
| 4 | 499 (9.8) | 333 (10) | 166 (8.7) |  |
| Coagulation, n (%) |  |  |  | 0.008 |
| 0 | 3,058 (60) | 1,865 (59) | 1,193 (63) |  |
| 1 | 902 (18) | 610 (19) | 292 (15) |  |
| 2 | 559 (11) | 352 (11) | 207 (11) |  |
| 3 | 364 (7.2) | 232 (7.3) | 132 (6.9) |  |
| 4 | 195 (3.8) | 118 (3.7) | 77 (4.1) |  |
| Liver, n (%) |  |  |  | <0.001 |
| 0 | 3,731 (73) | 2,265 (71) | 1,466 (77) |  |
| 1 | 594 (12) | 416 (13) | 178 (9.4) |  |
| 2 | 594 (12) | 387 (12) | 207 (11) |  |
| 3 | 93 (1.8) | 61 (1.9) | 32 (1.7) |  |
| 4 | 66 (1.3) | 48 (1.5) | 18 (0.9) |  |
| Cardiovascular, n (%) |  |  |  | 0.52 |
| 0 | 785 (15) | 505 (16) | 280 (15) |  |
| 1 | 862 (17) | 544 (17) | 318 (17) |  |
| 2 | 123 (2.4) | 72 (2.3) | 51 (2.7) |  |
| 3 | 1,488 (29) | 911 (29) | 577 (30) |  |
| 4 | 1,820 (36) | 1,145 (36) | 675 (36) |  |
| Neurology, n (%) |  |  |  | 0.67 |
| 0 | 2,926 (58) | 1,827 (58) | 1,099 (58) |  |
| 1 | 1,207 (24) | 745 (23) | 462 (24) |  |
| 2 | 341 (6.7) | 213 (6.7) | 128 (6.7) |  |
| 3 | 229 (4.5) | 153 (4.8) | 76 (4.0) |  |
| 4 | 375 (7.4) | 239 (7.5) | 136 (7.2) |  |
| Renal, n (%) |  |  |  | <0.001 |
| 0 | 1,881 (37) | 1,074 (34) | 807 (42) |  |
| 1 | 1,073 (21) | 728 (23) | 345 (18) |  |
| 2 | 935 (18) | 630 (20) | 305 (16) |  |
| 3 | 524 (10) | 323 (10) | 201 (11) |  |
| 4 | 665 (13) | 422 (13) | 243 (13) |  |
| ^1^Pearson's Chi-squared test for comparisons between categorical variables; Wilcoxon rank sum test for comparisons between continuous variables. | | | | |

**Supplementary Table 2**: Baseline characteristics stratified by sex for a sensitivity analysis excluding patients with ICU admission post operation or intervention.

ICU*intensive care unit, IMC intermediate care unit,* IQR*interquartile range,*NEMS*Nine Equivalents of Nursing Manpower Use Score,*LOS*length-of-stay,*RRT*renal replacement therapy,* SAPS II *Simplified Acute Physiology Score II, SAS sedation agitation scale, SOFA sequential organ failure assessment*

| **Variable** | **Overall**, N = 3,368 | **M**, N = 2,161 | **F**, N = 1,207 | **p-value**^1^ |
| --- | --- | --- | --- | --- |
| Admission diagnosis, n (%) |  |  |  | 0.91 |
| Sepsis | 1,260 (37) | 810 (37) | 450 (37) |  |
| Septic shock | 2,108 (63) | 1,351 (63) | 757 (63) |  |
| Age, Mean (SD) | 67.8 (13.9) | 68.2 (13.3) | 67.1 (15.0) | 0.44 |
| ICU admission from, n (%) |  |  |  | 0.47 |
| Other | 126 (3.7) | 83 (3.8) | 43 (3.6) |  |
| OtherICU | 167 (5.0) | 113 (5.2) | 54 (4.5) |  |
| IMC_RecoveryRoom | 184 (5.5) | 129 (6.0) | 55 (4.6) |  |
| EmergencyDepartment | 1,720 (51) | 1,094 (51) | 626 (52) |  |
| OR_Interv_Maternity | 111 (3.3) | 72 (3.3) | 39 (3.2) |  |
| Ward | 1,060 (31) | 670 (31) | 390 (32) |  |
| ICU discharge to, n (%) |  |  |  | 0.19 |
| Died | 500 (15) | 333 (15) | 167 (14) |  |
| External | 348 (10) | 233 (11) | 115 (9.5) |  |
| Within hospital | 2,520 (75) | 1,595 (74) | 925 (77) |  |
| Total NEMS normalized to shifts, Mean (SD) | 23.7 (6.6) | 23.8 (6.7) | 23.5 (6.3) | 0.25 |
| NEMS of first shift, Mean (SD) | 24.5 (8.3) | 24.8 (8.4) | 24.0 (7.9) | 0.014 |
| Mechanical ventilation, n (%) | 1,332 (40) | 876 (41) | 456 (38) | 0.12 |
| Vasoactive drug, n (%) | 2,576 (76) | 1,644 (76) | 932 (77) | 0.45 |
| Multiple vasoactive drugs, n (%) | 657 (20) | 447 (21) | 210 (17) | 0.021 |
| Renal replacement therapy, n (%) | 390 (12) | 255 (12) | 135 (11) | 0.59 |
| Sedation-agitation scale, n (%) | 388 (12) | 265 (12) | 123 (10) | 0.071 |
| Interventions on ICU, n (%) | 1,226 (36) | 812 (38) | 414 (34) | 0.058 |
| Non-invasive ventilation, n (%) | 2,873 (85) | 1,837 (85) | 1,036 (86) | 0.52 |
| Limitation of treatment, n (%) |  |  |  | 0.46 |
| None | 2,287 (68) | 1,477 (68) | 810 (67) |  |
| Admission | 603 (18) | 373 (17) | 230 (19) |  |
| Course | 429 (13) | 282 (13) | 147 (12) |  |
| Discharge | 49 (1.5) | 29 (1.3) | 20 (1.7) |  |
| Total SOFA on admission, Mean (SD) | 7.6 (3.6) | 7.7 (3.6) | 7.4 (3.6) | 0.006 |
| Max total SOFA, Mean (SD) | 8.6 (4.1) | 8.8 (4.0) | 8.4 (4.1) | <0.001 |
| SAPS, Mean (SD) | 47.2 (19.0) | 47.5 (19.0) | 46.7 (19.0) | 0.24 |
| ICU length of stay, Median (IQR) | 2.5 (1.2 – 5.1) | 2.6 (1.2 – 5.5) | 2.5 (1.2 – 4.8) | 0.23 |
| ICU mortality, n (%) | 500 (15) | 333 (15) | 167 (14) | 0.22 |
| ^1^Pearson's Chi-squared test; Wilcoxon rank sum test | | | | |

**Supplementary Table 3**: Selected baseline characteristics of patients without any form of advance directive in place. ICU*intensive care unit,*IQR*interquartile range, SD standard deviation, SOFA sequential organ failure assessment*

| **Characteristic** | **Overall**  N = 3,472 | **Men**  N = 2,185 | **Women**  N = 1,287 | **p-value**^1^ |
| --- | --- | --- | --- | --- |
| Age (years), Mean (SD) | 65.5 (14.4) | 66.3 (13.5) | 64.1 (15.7) | 0.001 |
| Total SOFA on admission, Mean (SD) | 7.3 (3.5) | 7.4 (3.5) | 7.1 (3.4) | 0.002 |
| ICU length of stay (days), Median (IQR) | 2.6 (1.3 – 5.6) | 2.6 (1.2 – 5.9) | 2.6 (1.4 – 5.1) | 0.43 |
| ICU mortality, n (%) | 221 (6.4) | 147 (6.7) | 74 (5.7) | 0.25 |
| ^1^Pearson's Chi-squared test for comparisons between categorical variables; Wilcoxon rank sum test for comparisons between continuous variables. | | | | |

**Supplementary Figure 1**: Patient flow chart


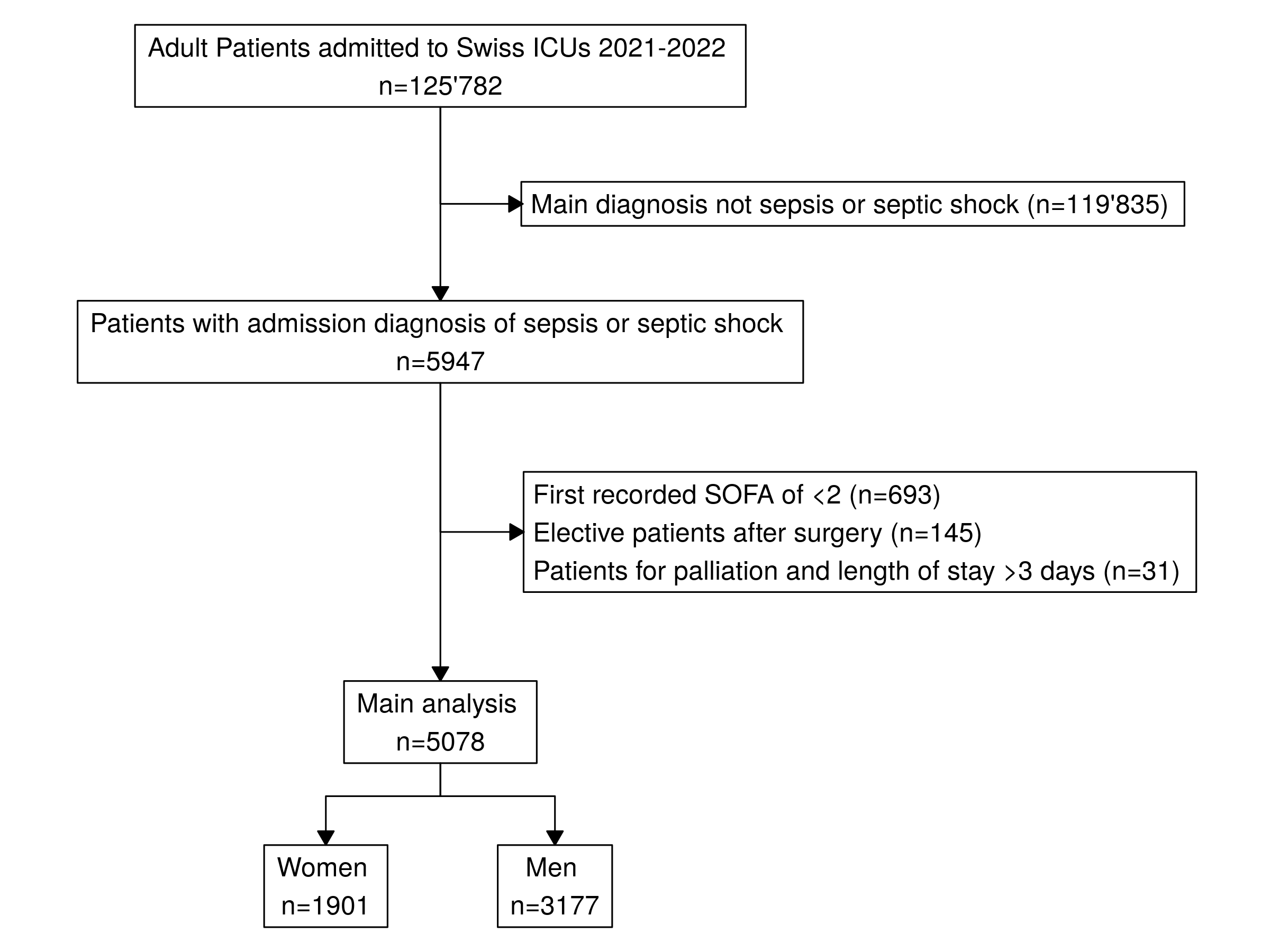


**Supplementary Figure 2:** The distribution plot **(A)** shows a tendency towards lower maximum SOFA scores in women compared to men within the first 21 days of ICU stay. There were no notable differences in the time from admission to the maximum recorded SOFA score **(B)** between sexes, which in most patients was present at admission or shortly thereafter. *SOFA Sequential Organ Failure Assessment*


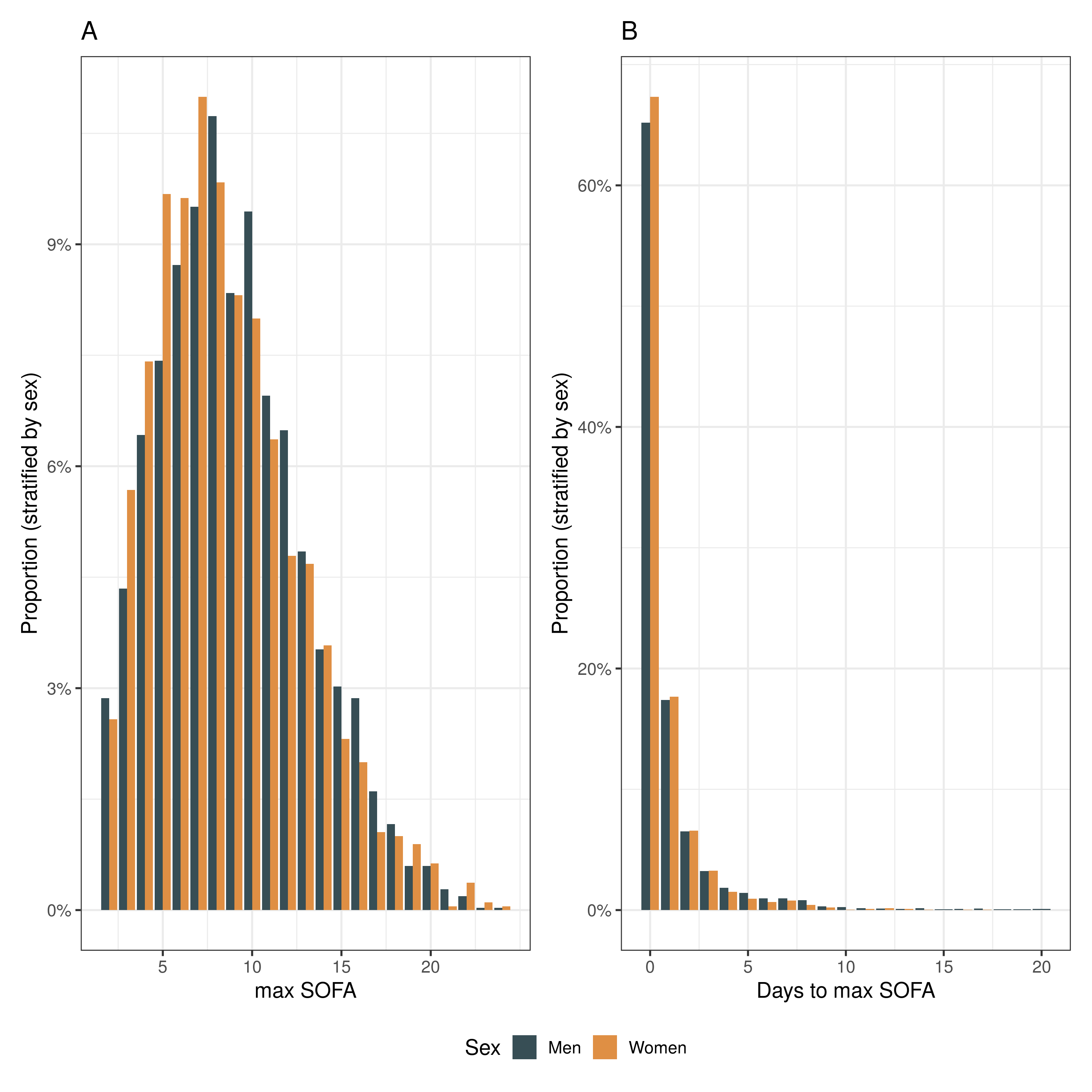


**Supplementary Figure 3:** Distribution of SOFA components stratified by sex and age 52. Distributions are mostly skewed with a predominance of lower scores with the cardiovascular and respiration components being the exception. Notable differences between women and men can be observed in the coagulation, liver and renal components, which show a higher proportion of women scoring 0 points. Differences between sexes tend to be greater in younger patients <52 years. *SOFA* *Sequential Organ Failure Assessment*


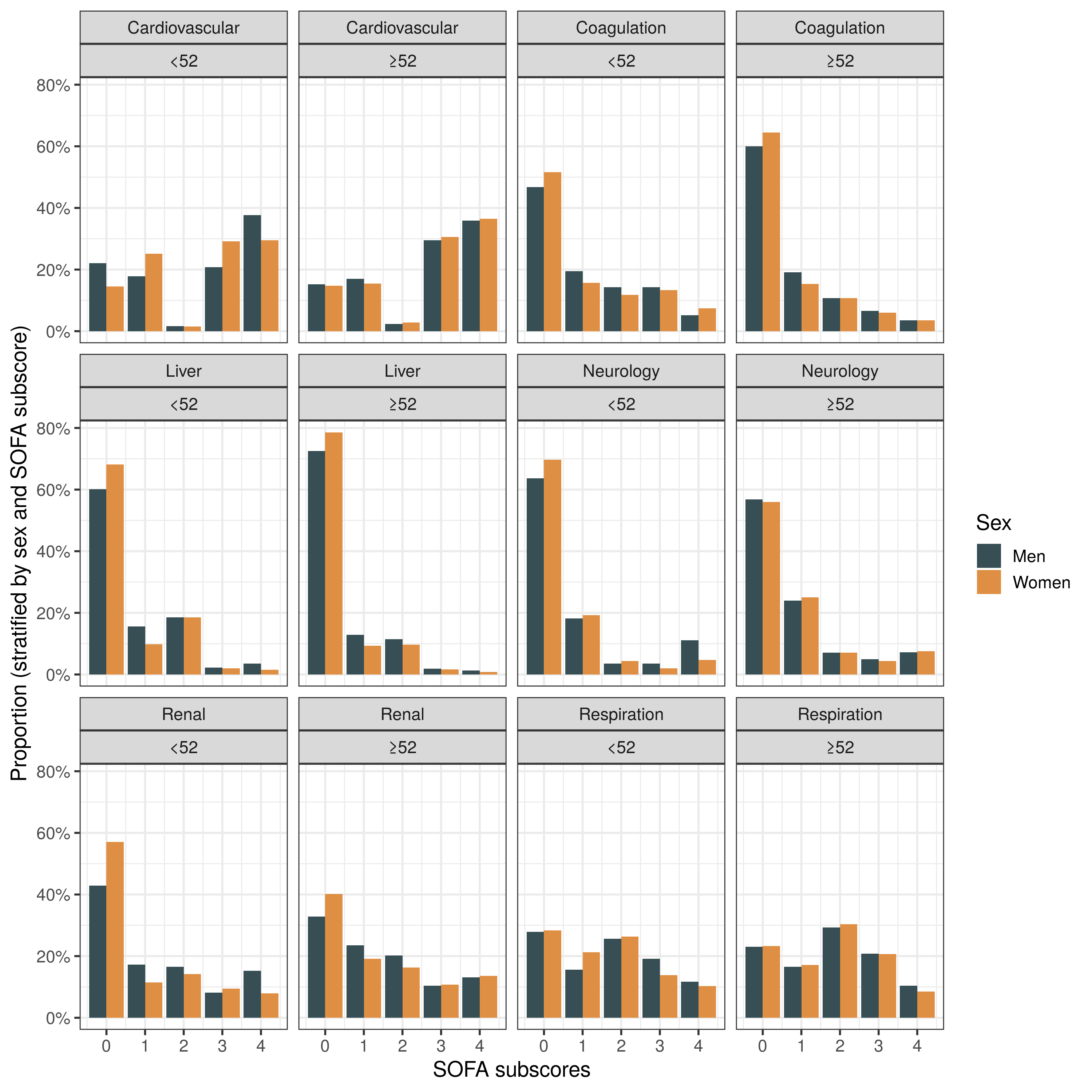


**Supplementary Figure 4**: Conditional effect plots of a logistic regression model where the dependent variable is ICU death, and independent variables include total SOFA score, age and sex **(A)**. To relax linearity assumptions, age and total SOFA were modeled using restricted cubic splines (4 knots each). **B** shows conditional effect plots of the model including age and sex as well as the SOFA sub-scores. Of the SOFA sub-scores, the nervous and respiratory system sub-scores have the steepest ascent and show the strongest effect on the outcome. In both models, sex did not have a relevant impact on model performance. *SOFA* *Sequential Organ Failure Assessment.*


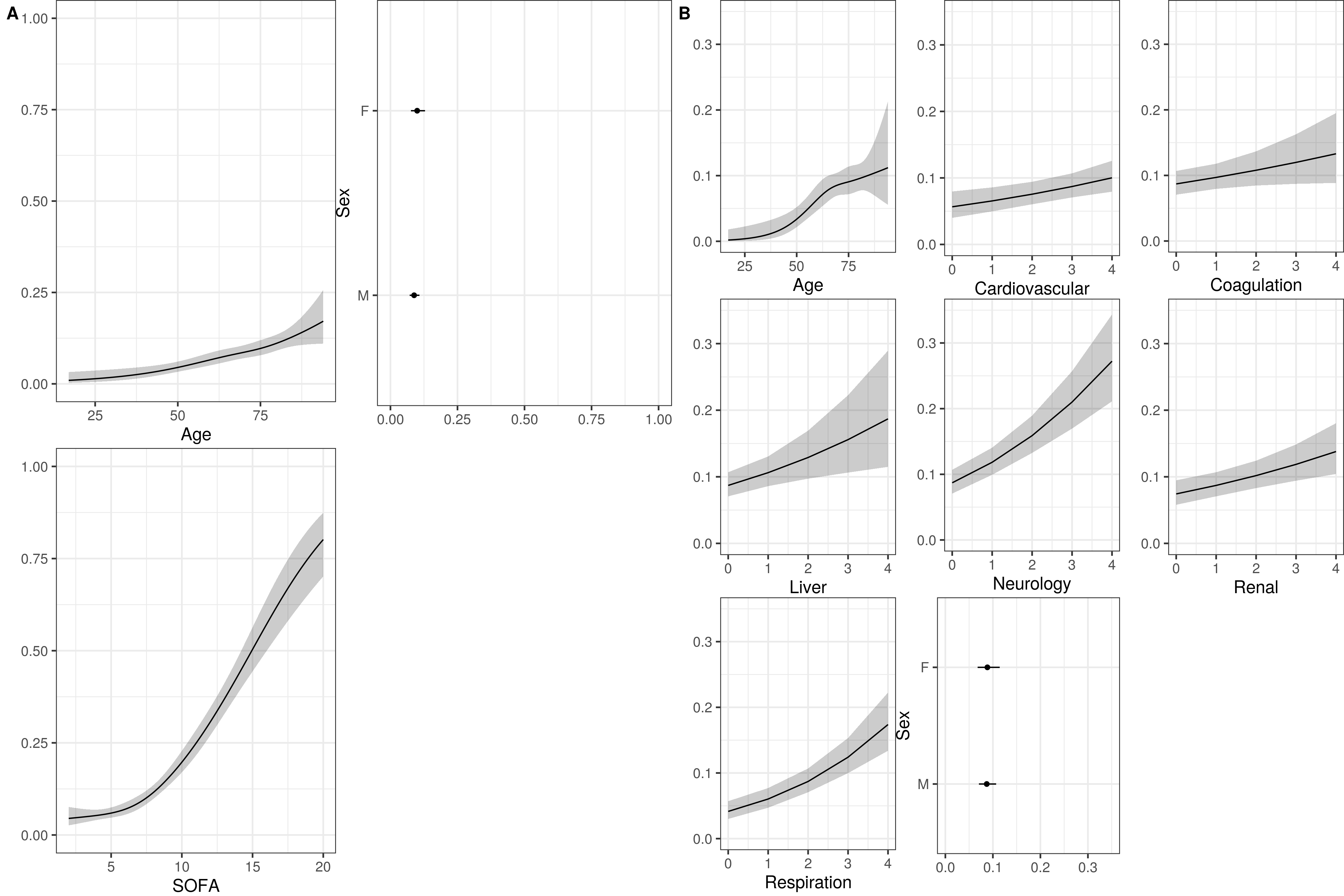


**Supplementary Figure 5:** ANOVA plot of SOFA components, sex and age in a logistic regression model with ICU death as outcome, displaying the importance of each variable in the model. The respiration and neurology components, together with age, have the greatest influence on model predictions.


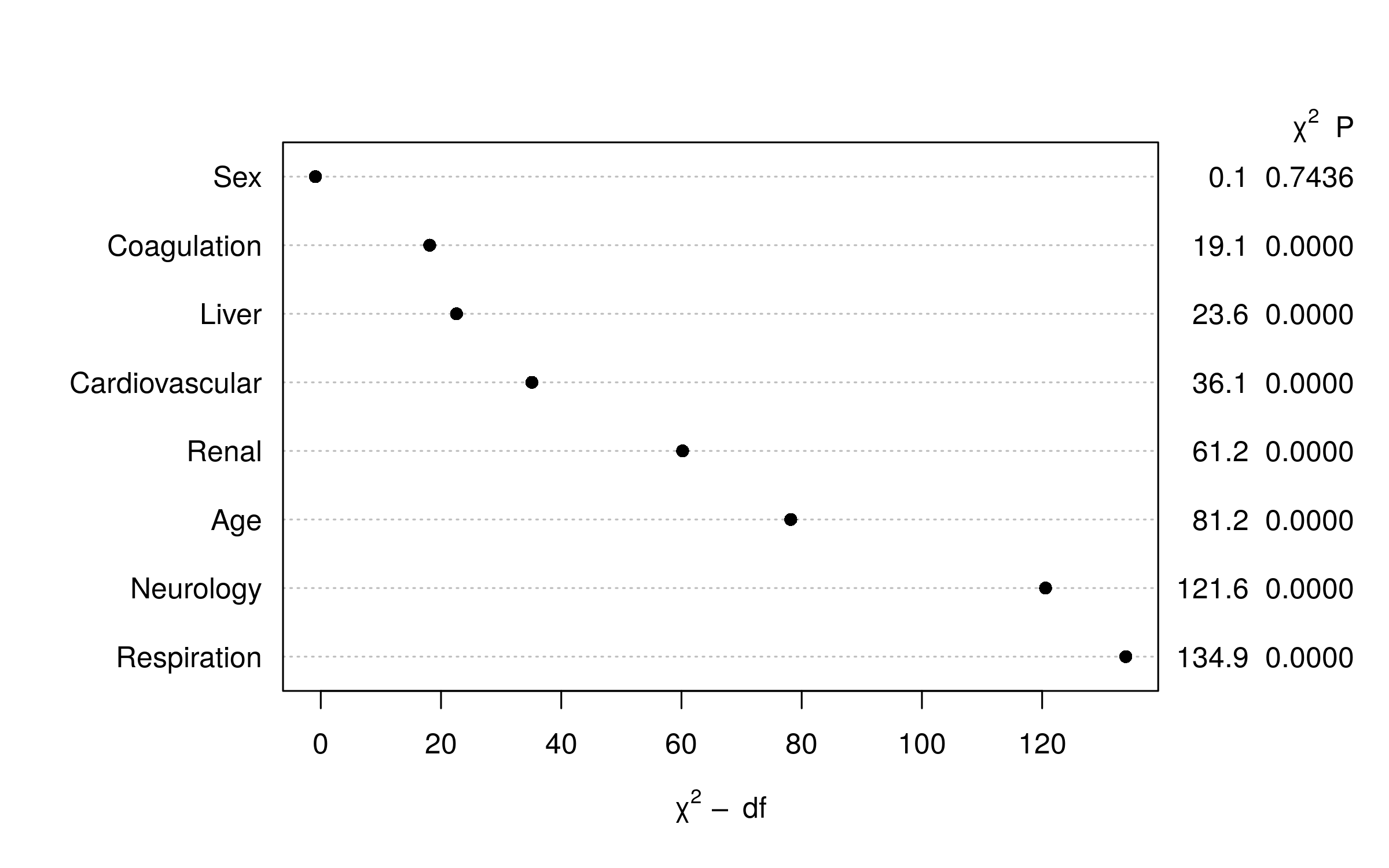


**Supplementary Figure 6***:* Quantile regression to the median showing sex-specific differential associations between age and total SOFA. SOFA scores increase in men with increasing age until around 60 years and a downward trend afterwards, while an increase in total SOFA with age can be observed in women across the entire age spectrum. *SOFA Sequential Organ Failure Assessment*


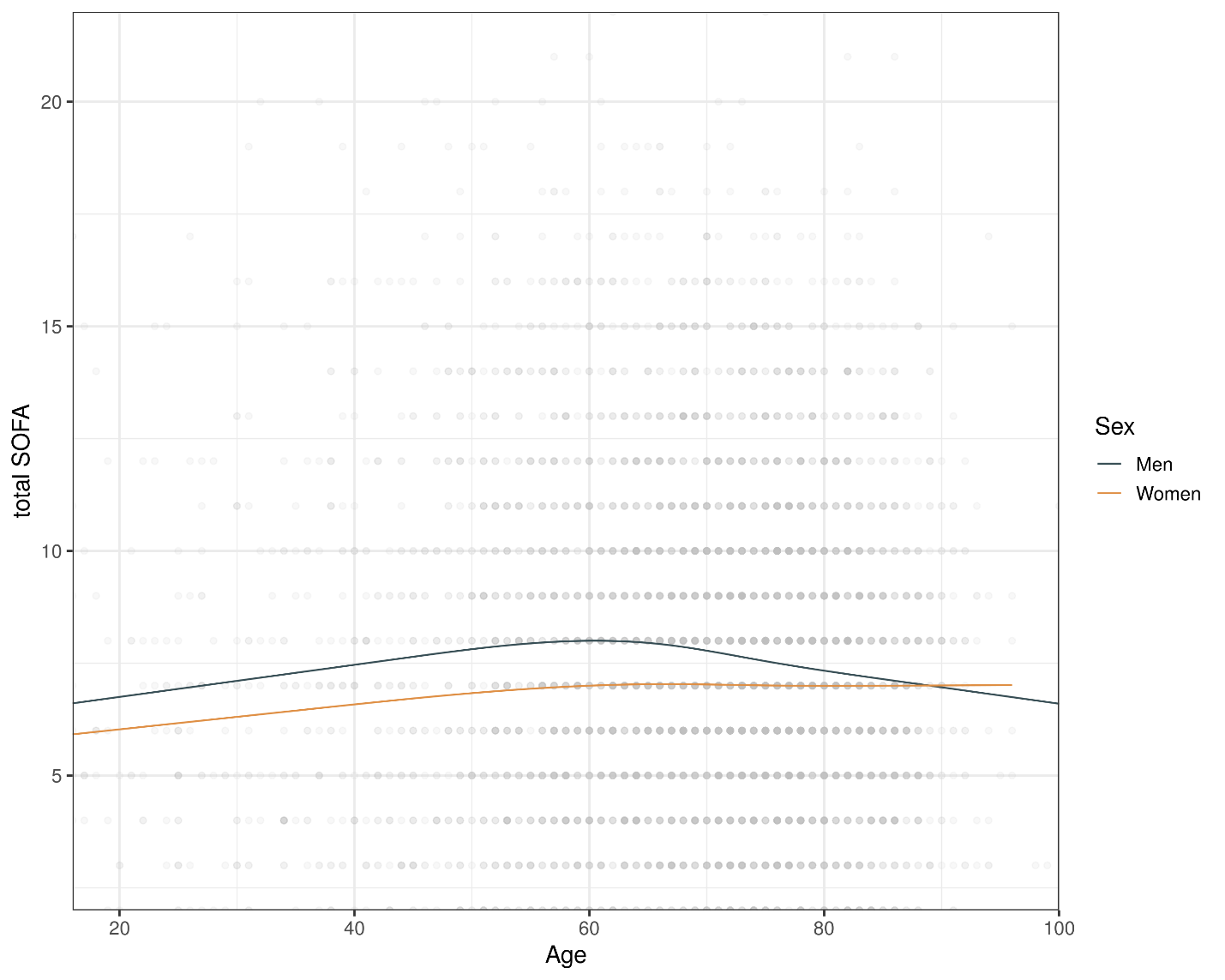

Supplement: Supplementary file 2 — Supplementary Material 2. [file 13054_2024_4996_MOESM2_ESM.docx]
